# Case Report: Rare Pulmonary Sclerosing Pneumocytoma: Large, Multiple, Metastatic, and Fatal

**DOI:** 10.3389/fmed.2021.661032

**Published:** 2021-08-16

**Authors:** Weidong Zhang, Yuanyuan Liu, Yamei Chai, Kefeng Shi, Jialing Chen, Dongfeng Shi, Xiaoming Wu

**Affiliations:** ^1^Department of Thoracic Surgery, Henan Provincial Chest Hospital, Zhengzhou, China; ^2^Department of Pathology, Henan Provincial Chest Hospital, Zhengzhou, China

**Keywords:** pulmonary sclerosing pneumocytoma, large, multiple, metastases, fatal

## Abstract

Pulmonary sclerosing pneumocytoma (PSP) is a rare benign or low-grade malignant tumor, but it has the potential to present with multiple lesions, lymph node metastasis, extra-pulmonary metastasis, recurrence and even cause death. Herein, a case of PSP that was huge, presented with multiple lesions and had lymph node as well as extrapulmonary metastases (liver, abdominal cavity, bones) is reported for the first time. This patient was also the first one to die of respiratory and circulatory failure caused by the PSP tumor and its metastases which compressed the mediastinal tissue.

## Introduction

In 2015, the World Health Organization (WHO) officially renamed Pulmonary Sclerosing Hemangioma (PSH) to Pulmonary Sclerosing Pneumocytoma (PSP) and classified it as a lung adenoma (1). Notably, PSP mostly occurs in Asian women (2), has low overall incidence, which has gradually increased over the recent years. In addition, PSP generally develops slowly, showing no progress for many years and most patients display no obvious symptoms during medical examination (3, 4). Moreover, previous research (5, 6) showed that PSP is a single and benign tumor although some studies reported that it can present with multiple lesions and metastases, including lymph nodes (7, 8), other lungs (9), liver (10), bone (11), pleura (12), and stomach (13), or even recurrence (14). Similarly, the present article reports on a patient that had a large PSP tumor with several lesions, vascular invasion and multiple metastases in the mediastinal lymph nodes, liver, celiac lymph nodes, and bones. However, the patient eventually died of PSP.

## Case Presentation

**Figure 1:**
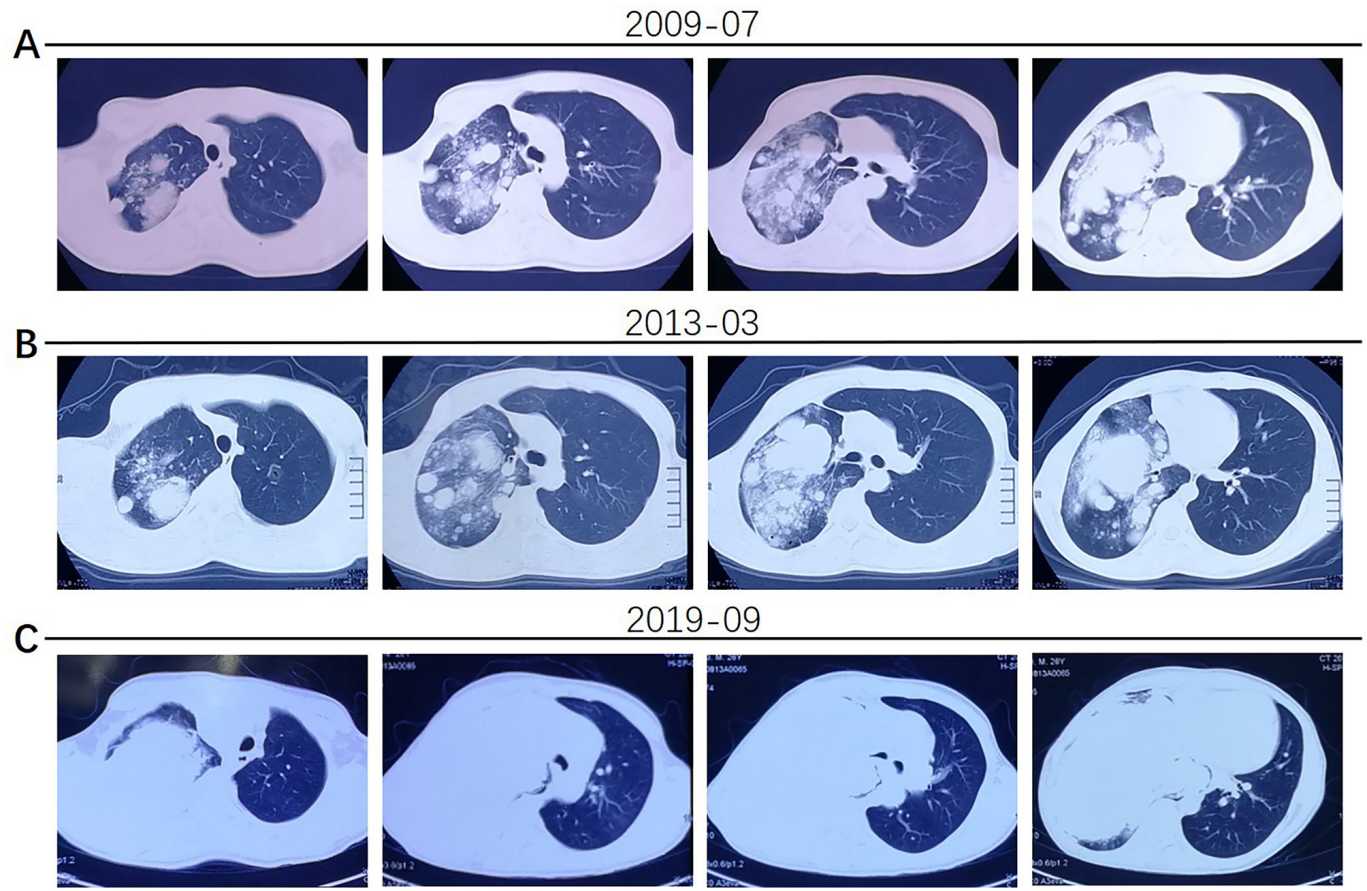
**(A)** The patient's chest CT examination conducted in 2009 showed multiple nodular shadows in the right lung. **(B)** The 2013 chest CT examination showed larger, multiple shadows in the right lung. **(C)** In 2019, the patient had worse chest tightness and his chest CT scan showed a large irregular mass in the right lung, multiple nodules of varying sizes in the surrounding lung and invasion of the superior vena cava, right pulmonary artery and vein.

**Figure 2:**
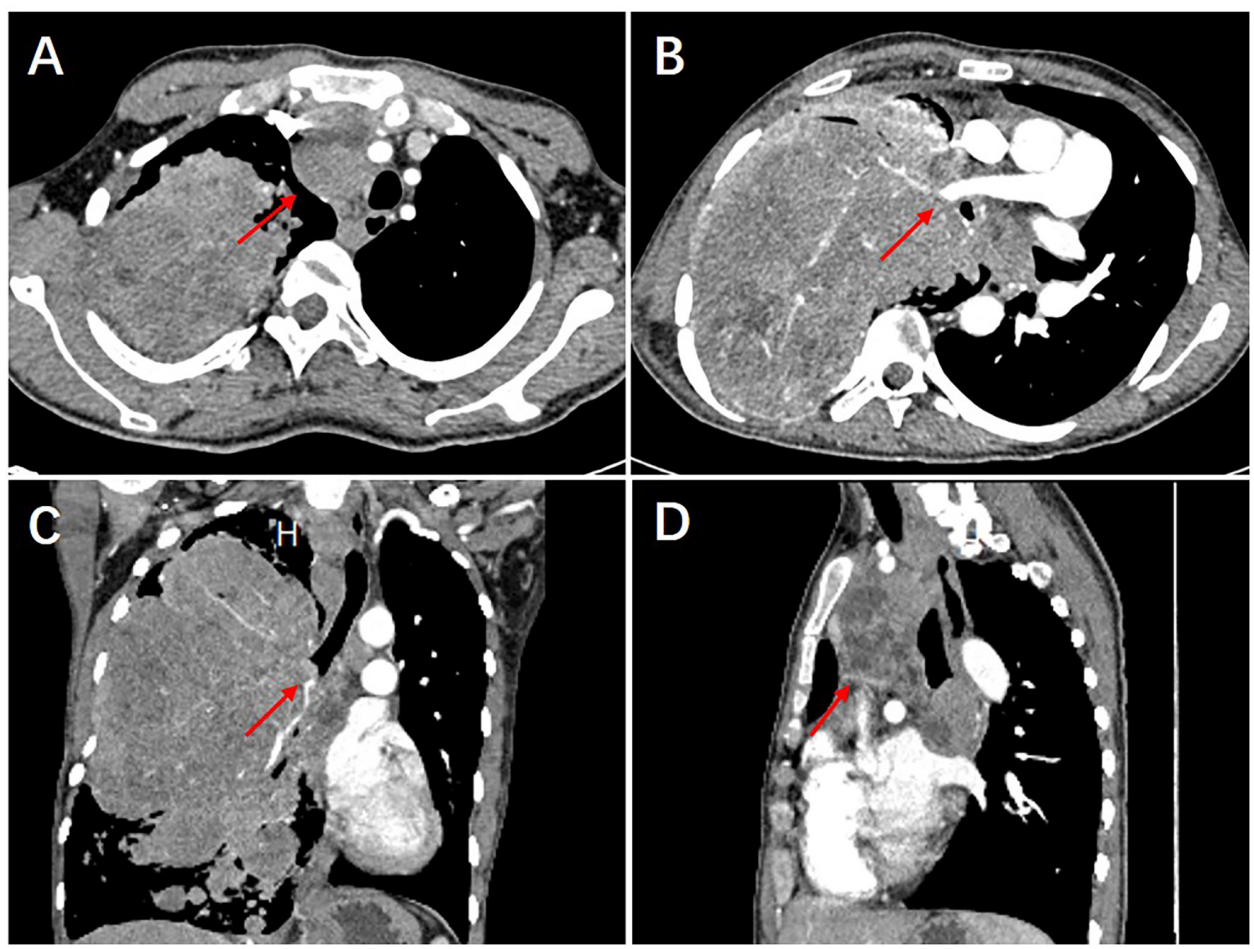
The 2019 chest CT examination showed an irregular large mass in the right lung, about 226 × 145 × 143 mm in size, with calcification and some low-density areas. The scan showed uneven enhancement, after injection of the contrast medium. Multiple nodules of varying sizes were also seen in the lungs surrounding the mass **(A)**. The superior vena cava, some branches of the right pulmonary artery and the right pulmonary vein were also all invaded **(B)**. In addition, bronchial stenosis in the upper, middle, and lower lobes of the right lung was observed **(C)**. The mediastinum was shifted to the left due to compression and multiple enlarged lymph nodes were seen in the mediastinum **(D)**.

The patient was a 25-year-old male who was admitted to our hospital (Henan Provincial Chest Hospital) in August 2019 with “a mass in the right lung, which had persisted for more than 10 years and had chest tightness as well as shortness of breath for more than 1 month.” In July 2009, the patient presented in a local hospital due to “fatigue” and chest Computed Tomography (CT) showed a shadow of multiple masses in the right lung, as shown in Figure 1A, but he was never treated. In March 2013, due to physical examination, the patient underwent a second chest CT (Figure 1B) which revealed a larger shadow of masses in the right lung, compared to the one seen 4 years before. Additionally, CT-guided lung biopsy revealed alveolar epithelial cell hyperplasia and local tissue papillary hyperplasia, indicating possible diagnosis of PSP. The attending doctor then recommended a right pneumonectomy, but the patient refused to receive any further treatment. In July 2019, the patient experienced chest tightness and shortness of breath, accompanied by coughing and hemoptysis. Re-examination through chest CT (Figures 1C, 2) revealed a large irregular mass in the right lung, with calcification and some low-density areas. Moreover, injection of the contrast medium showed uneven enhancement and multiple nodules of varying sizes surrounding the mass in the lung. The superior vena cava, some branches of the right pulmonary artery and the right pulmonary vein were also all invaded. Notably, the lesions were larger, compared to those observed in the chest CT performed in 2013. Furthermore, bronchoscopy revealed that the bronchial openings in the upper, middle, and lower lobes of the right lung were narrowed and fish-like tissues were seen at the opening of the dorsal section of the right lung, blocking the bronchial lumen. Thereafter, a biopsy of the diseased tissue was obtained and microscopic examination revealed a mass of atypical cells. Additionally, immunohistochemistry analysis suggested papillary adenoma and the patient was advised to have a larger tissue biopsy.

**Figure 3:**
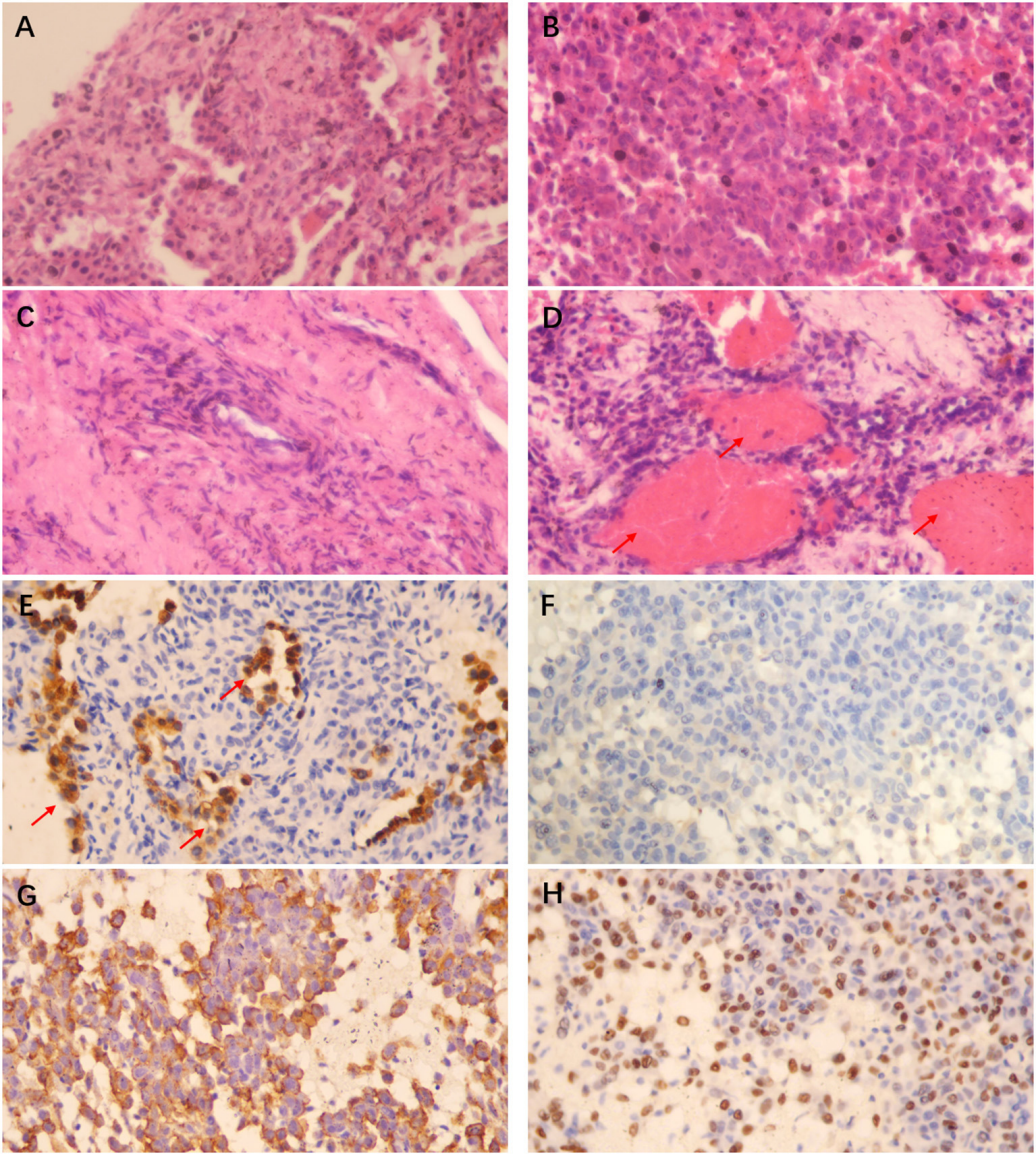
HE staining (×400) showing that the papillary area was composed of many cuboidal surface cells and rounded cells **(A)**, the solid area was mainly composed of rounded cells of similar size **(B)**, focal hyperplasia could be found around the vascular wall in the sclerosing area **(C)**, and a large number of red blood cells could be observed filling the lung interstitium and alveolar cavity in the hemorrhagic area (shown by the arrow in **D**); Histochemistry (×400) shows that the cuboidal surface cells of the tissue are positive for Pan-cytokeratin (Pan-CK) (shown by the arrow in **E**), while the rounded cells are negative for Pan-CK **(F)**, positive for Epithelial Membrane Antigen (EMA) **(G)** and Thyroid Transcription Factor-1 (TTF-1) **(H)**. The pathological manifestations are consistent with the diagnosis of PSP.

Although the chest CT suggested that the patient most likely had a malignant tumor in the lung, a clear pathological diagnosis was not made even after several medical examinations. Subsequently, a CT-guided lung biopsy was performed, where the tumor tissue was punctured multiple times at different tumor sites and the pathological diagnosis was confirmed to be PSP (Figure 3). It is noteworthy that these pathological findings were confirmed by different chief pathologists, in order to further verify the diagnosis.

**Figure 4:**
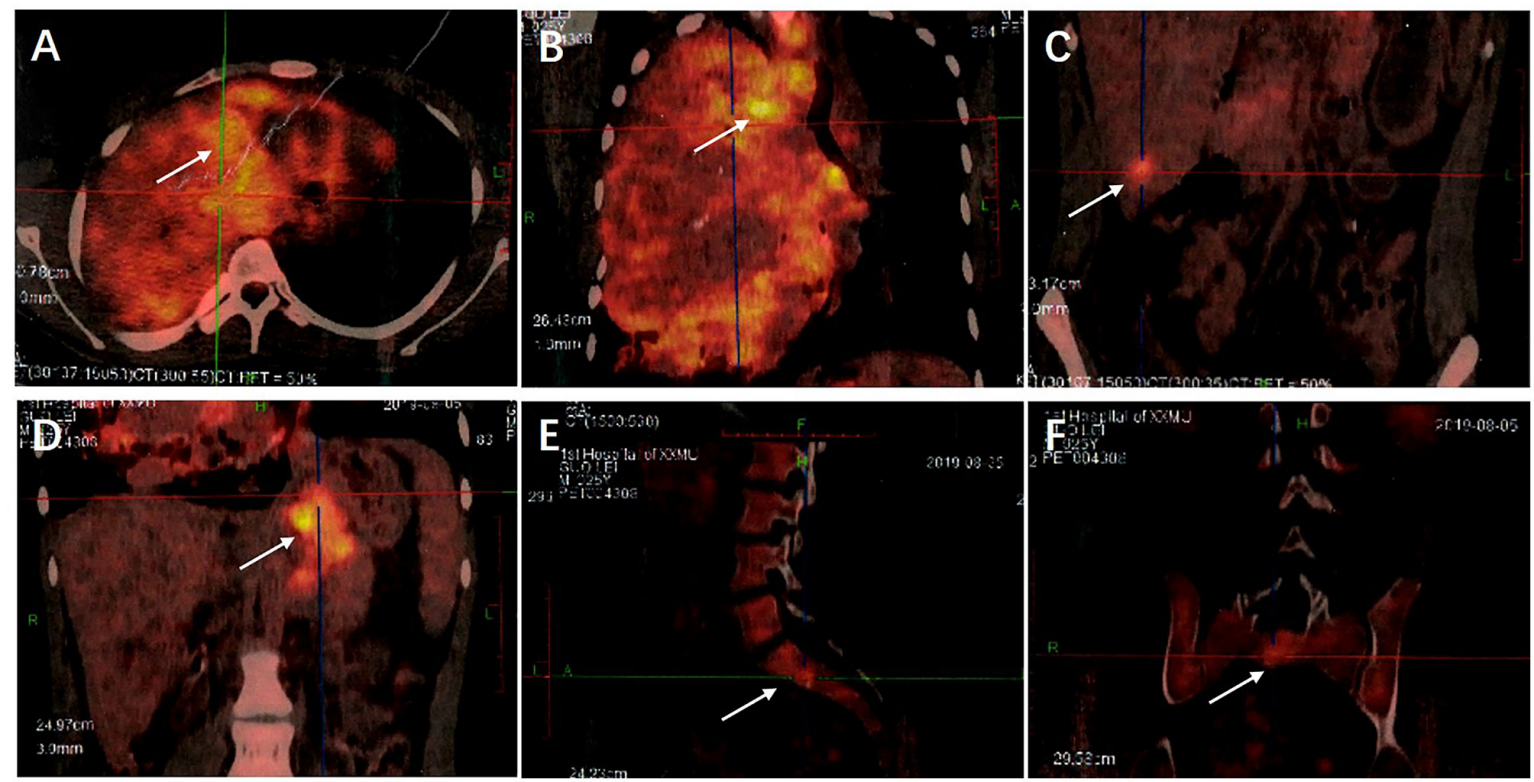
**(A)** The 18F-FDG PET/CT examination showed a huge irregular mass in the right lung with uneven density and increased FDG uptake. The SUVmax was 8.21. **(B)** Multiple enlarged lymph nodes were seen in the 2R, 2L, 4R, 4L, 5, 7, 8, and 10R areas of the mediastinum, their FDG intake had unevenly increased and the SUVmax was 10.39. **(C)** Round low-density nodules were present in the lateral segment of the left lobe of the liver and the posterior lower segment of the right lobe of the liver, respectively. Round low-density nodules were also present in the left and right lobes of the liver, respectively, with unclear borders and increased FDG uptake. The SUVmax was 4.48. **(D)** Multiple enlarged lymph nodes were present in the abdominal cavity, there was an increase in FDG uptake and the SUVmax was 6.79. **(E,F)** Low-density lesions were seen in the lumbar 1 and sacral 1 vertebral bodies, FDG uptake had increased and the SUVmax was 4.58. Examination through 18F-FDG PET/CT showed a malignant tumor in the right lung with multiple metastases in the hilar and mediastinal lymph nodes, several bones, liver, and intra-abdominal lymph nodes.

Additionally, Fluorodeoxyglucose Positron Emission Tomography/Computed Tomography (18F-FDG PET/CT) examination during hospitalization showed a malignant tumor in the right lung, with multiple metastases in the hilar and mediastinal lymph nodes, several bones, liver, and intra-abdominal lymph nodes (Figure 4).

Color Doppler echocardiography also revealed mild pulmonary hypertension (systolic pressure 43 mmHg, simplified Bernoulli equation) and pericardial effusion, about 10 mm from the top of the right atrium.

Laboratory examination revealed that the level of Hemoglobin (HGB) was 84 g/L (120-160 g/L), the Red Blood Cell (RBC) count was 4.21 × 10^12^/L (4-5.5/L), Hematocrit (HCT) was 0.285 (0.36-0.5), white blood cell count was 8.6 × 10^9^/L (4 ~ 10 × 109/L) and the percentage of neutrophils was 76.3% (50 ~ 70%). In addition, the concentration of glutamic pyruvic transaminase was (ALT) 41.2 U/L (0 ~ 40 U/L), that of glutamic oxaloacetic transaminase was (AST) 16.5 U/L (0 ~ 40 U/L), Total Protein (TP) concentration was 56.8 g/L (60.0 ~ 85.0 g/L), albumin concentration was (ALB) 21.6 g/L (35.0 ~ 55.0 g/L) and Globulin (GLOB) concentration was 35.2 g/L (20.0 ~ 35.0 g/L). Moreover, the patient had normal concentrations of tumor markers such as the Prostate Specific Antigen (PSA), Carcinoembryonic Antigen (CEA), Alpha-fetoprotein (AFP), cytokeratin 19 fragment, Neuron-specific Enolase (NSE), and Carbohydrate Antigen 125 (CA125).

Moreover, whole-exome sequencing was performed on the patient's PSP tumor tissue and the matched normal sample from the peripheral blood was analyzed through the Switching Mechanism at the 5' end of RNA Transcripts (SMART) method. Notably, activation of the AKT1- p.E17K pathway was observed in 31 genes (AKT1, ALK, APC, ATM, BRAF, BRCA1, BRCA2, EGFR, ERBB2, FGFR1, FGFR2, FGFR3, HRAS, KIT, KRAS, MAP2K1, MAP2K2, MET, MTOR, NRAS, NTRK1, NTRK3, PDGFRA, PIK3CA, PTEN, RET, ROS1, SMAD4, TP53, TSC1, and TSC2).

After hospitalization, physical examination showed that the patient was emaciated, had poor mental health, assumed a semi-recumbent position and had mild shortness of breath. The patient's temperature was 36.6°C, his pulse was 108 bpm, respiratory rate was 30 breaths per minute and blood pressure was 128/71 mmHg. In addition, a 15 cm surgical incision scar was seen on the right thorax and the chest cavity was symmetrical without deformity. Lung auscultation also revealed no respiratory sound on the right lung and the left lung was normal.

Notably, the patient had undergone “right pulmonary bulla resection” surgery for the right pneumothorax, 19 years earlier and had no history of other diseases.

Furthermore, the patient was diagnosed with: (1) Malignant PSP of the right lung with multiple metastases in the blood vessels, mediastinum, liver, bone, and celiac lymph nodes; (2) Hypoproteinemia; (3) Anemia; (4) Pulmonary hypertension; and (5) Pericardial effusion.

After admission, the patient was placed on oxygen, administered with supplements and received supportive treatment. His symptoms improved slightly following this treatment. However, his physical condition was not suitable for surgical treatment and he chose to leave the hospital. Six months later, the patient died of breathing difficulties and cachexia.

## Discussion

PSP is a rare tumor with unique histological characteristics, which can be summarized as “Two cell types, four patterns.” In addition, PSP typically comprises of a mixture of four architectural patterns (papillary, solid, sclerosing, and hemorrhagic) and two cell types, namely; cuboidal surface cells and rounded cells with either an eosinophilic or clear cytoplasm (15, 16). The disease is also more common in middle-aged Asian females (2–4). Moreover, numerous studies (7–14) showed that PSP is a rare benign or low-grade malignant tumor, with the potential of lymph node metastasis, extra-pulmonary metastasis, multiple lesions, and recurrence. However, no fatal cases have been reported so far. Therefore, this was the first case of PSP characterized by multiple lesions which were large and at the same time metastasized to the lymph nodes and extra-pulmonary regions (liver, abdominal cavity, and multiple bones). This was also the largest mass of PSP ever observed and was accompanied by vascular invasion. Furthermore, this was the first patient ever reported to have died of respiratory failure and cachexia caused by the PSP tumor.

In 1986, the Japanese scholars, Tanaka et al. (17) and Spencer and Nambu (18) reported that a 22-year-old male patient diagnosed with sclerosing hemangioma (now referred to as PSP) underwent lobectomy and lymph node dissection. However, hilar lymph node metastasis was inadvertently observed during pathological examination. This was the first reported case of PSP with lymph node metastasis. Since then, there have been more reports of lymph node metastasis in PSP although extra-pulmonary metastasis is rarely reported. For example, Li et al. (a Chinese scholar) (12) reported on a case of PSP with pleural metastasis in 2006 while Bae et al. (a South Korean scholar) (13) reported on a PSP patient with gastric metastasis in 2012. Additionally, Kim et al. (a South Korean scholar) (19) reported on a case of PSP with L3 vertebral metastasis in 2015. In 2017, Zhan et al. (a Chinese scholar) (10) reported on a case of PSP accompanied with liver metastasis and in 2019, Lim et al. (a South Korean scholar) (11) reported on a PSP patient with T5 vertebral metastasis.

Currently, the mechanism of PSP metastasis in patients is not yet fully understood although a few suggestions have been put forward. For instance, Suzuki et al. (20) suggested that increased expression of Matrix Metalloproteinase-9 (MMP-9) plays an important role in the occurrence and metastasis of PSP. On the other hand, Wang et al. (21) and Teng et al. (22) suggested that round cells may originate from Epithelial-mesenchymal Transformation (EMT) of surface cells and may be closely related to tumor metastasis. Additionally, Jiang et al. (23) conducted Sanger sequencing and Next-generation Sequencing (NGS) on samples from a patient with multiple PSP and found a BRAF V600E mutation and the activation of the AKT1-E17K pathway, which was speculated to be related to the malignant transformation of PSP. Moreover, Fan et al. (24) assessed somatic mutations through sequencing and also conducted pathway analysis. The results showed that the activation of AKT1 may also play a role in the occurrence and development of PSP, similar to the present study where the AKT1-p.E17K pathway was also activated. Although previous research suggested that the occurrence and metastasis of PSP may be related to the activation of the AKT1-p.E17K pathway, the mechanism of metastasis and malignancy is still unclear.

Before the patient was diagnosed with PSP in this case, it was speculated that he had adenoma. This may have been due to the limited specimen and at the time, only the papillary area was observed. However, repeated puncture of the lung lesions under CT-guided lung biopsy provided a large amount of tumor tissue and a final diagnosis of PSP was achieved. In addition, analysis of the chest CT images revealed multiple metastases in the mediastinal tissue, liver, abdominal cavity, bone, lymph nodes, and even vascular invasion. Examination through 18F-FDG PET/CT also showed a huge mass in the right lung. Additionally, there was an increase in FDG uptake and the Standardized maximum Uptake Value (SUVmax) was 8.21. There was also an increase in FDG uptake by multiple nodules in the remaining lobe of the right lung and the SUVmax was 3.91. Moreover, the hilar and mediastinal lymph nodes were enlarged, FDG uptake had increased unevenly and the SUVmax was 10.39. Multiple round nodules were also seen in the liver, the boundary was not clear, FDG uptake had increased and the SUVmax was 4.48. Additionally, both the L11 and S1 vertebrae had small flaky low-density lesions, there was an increase in FDG uptake and the SUVmax was 4.58. Furthermore, there were nodular FDG high-uptake areas on the left scapula, left iliac bone and left the ischial body, respectively, and the SUVmax was 3.67. There were also multiple, enlarged lymph nodes in the abdominal cavity, FDG uptake had increased and SUVmax was 6.79. These lesions indicated a significant increase in SUV uptake. Notably, given the patient's medical history, main diagnosis and information from previous literature (25, 26), the present study found no need to conduct pathological biopsy for each lesion, to confirm the diagnosis. In addition, the patient could also not tolerate multiple pathological biopsies to confirm diagnosis of each tissue.

Currently, no study has reported on PSP related deaths and most scholars still believe that PSP is a benign lesion with good prognosis and is not fatal (27, 28). However, PSP patients can have their lung tissue destroyed and surrounding blood vessels invaded. In addition, their trachea and heart may be harmed and this may affect cardiac function, cause pericardial effusion and lead to pulmonary hypertension. All these effects are associated with the growth of tumor lesions, resulting in respiratory and circulatory dysfunction, which may be accompanied by severe malnutrition and the patient finally dies of exhaustion.

In summary, this was the first reported case of PSP that presented with multiple lesions, was large and had lymph node as well as extrapulmonary metastases (liver, abdominal cavity, and multiple bones). The tumor had the largest diameter ever reported and was accompanied by vascular invasion. This was also the first patient to die of respiratory and circulatory failure caused by the PSP tumor and metastases compressing the mediastinal tissue. This outcome differed from previous reports on PSP and therefore requires more attention from healthcare practitioners. Moreover, further research is required to identify more clinical features and mechanisms of metastasis in PSP.

## Ethics Statement

Written informed consent was obtained from the individual(s) for the publication of any potentially identifiable images or data included in this article.

## Author Contributions

WZ and XW designed the entire study. WZ, YL, and DS conducted patient clinical management and sample collection. JC, YC, and KS analyzed the data. WZ wrote the manuscript. All the authors read and approved the final version of the manuscript for submission.

## Conflict of Interest

The authors declare that the research was conducted in the absence of any commercial or financial relationships that could be construed as a potential conflict of interest.

## Publisher's Note

All claims expressed in this article are solely those of the authors and do not necessarily represent those of their affiliated organizations, or those of the publisher, the editors and the reviewers. Any product that may be evaluated in this article, or claim that may be made by its manufacturer, is not guaranteed or endorsed by the publisher.

## References

[1] Travis WD Brambilla E Nicholson AG Yatabe Y Austin JHM Beasley MB. The 2015 world health organization classification of lung tumors: impact of genetic, clinical and radiologic advances since the 2004 classification. J Thorac Oncol. (2015) 10:1243–60. 10.1097/JTO.0000000000000630 26291008

[2] Chen B Gao J Chen H Cao Y He X Zhang W. Pulmonary sclerosing hemangioma: a unique epithelial neoplasm of the lung (report of 26 cases). World J Surg Oncol. (2013) 11:85. 10.1186/1477-7819-11-85 23587094 PMC3636073

[3] Khoo AC Hamzah F Ong CK. Incidental sclerosing pneumocytoma detected on bone scintigraphy. Clin Nucl Med. (2017) 42:e77-9. 10.1097/RLU.0000000000001371 27749415

[4] Zhou J Covinsky MH. Sclerosing pneumocytoma: a carcinoma mimicker. a case report and literature review. Ann Clin Lab Sci. (2017) 47:103–5. 28249927

[5] Devouassoux-Shisheboran M Hayashi T Linnoila RI Koss MN Travis WD. A clinicopathologic study of 100 cases of pulmonary sclerosing hemangioma with immunohistochemical studies: TTF-1 is expressed in both round and surface cells, suggesting an origin from primitive respiratory epithelium. Am J Surg Pathol. (2000) 24:906–16. 10.1097/00000478-200007000-00002 10895813

[6] Hu AM Zhao D Zheng H Wang QH Lyu Y Li BL. Preoperative diagnosis in 46 cases of pulmonary sclerosing hemangioma. Chin Med J (Engl). (2016) 129:1377–8. 10.4103/0366-6999.182839 27231179 PMC4894052

[7] Soo IX Sittampalam K Lim CH. Pulmonary sclerosing pneumocytoma with mediastinal lymph node metastasis. Asian Cardiovasc Thorac Ann. (2017) 25:547–9. 10.1177/0218492317727668 28825313

[8] Xu HM Zhang G. A rare case of pulmonary sclerosing hemagioma with lymph node metastasis and review of the literature. Int J Clin Exp Pathol. (2015) 8:8619–23. 26339444 PMC4555772

[9] Komatsu T Fukuse T Wada H Sakurai T. Pulmonary sclerosing hemangioma with pulmonary metastasis. Thorac Cardiovasc Surg. (2006) 54:348–9. 10.1055/s-2005-872976 16902885

[10] Zhan XY Wang JQ Zhou JX Li QJ Wang ZZ Han F. Pulmonary sclerosing hemangioma with liver metastasis: report of one case and literature review. Chin J Hepat Surg(Electronic Edition). (2017) 6:50–3. 10.3877/CMA.J.ISSN.2095-3232.2017.01.011

[11] Lim B Jeon W Han SW Kwack WG Lee SH. A case of pulmonary sclerosing pneumocytoma with multiple lung and bone metastasis. Am J Respir Crit Care Med. (2019) 199:A6950. 10.1164/ajrccm-conference.2019.199.1_MeetingAbstracts.A6950

[12] Li J Lu Q Jie J. Mediastinal lymph node and pieura metastasis of pulmonary sclerosing hemangioma: case report. Chin J Med Imaging Technol. (2006) 22:815. 10.1007/s11769-006-0026-1

[13] Bae YS Ro JY Shim HS Hong SW Yoon SO. Pulmonary sclerosing haemangioma with metastatic spread to stomach. Histopathology. (2012) 60:1162–4. 10.1111/j.1365-2559.2012.04213.x 22394410

[14] Iyoda A Hiroshima K Shiba M Haga Y Moriya Y Sekine Y. Clinicopathological analysis of pulmonary sclerosing hemangioma. Ann Thorac Surg. (2004) 78:1928–31. 10.1016/j.athoracsur.2004.05.069 15561002

[15] Nicholson AG Magkou C Snead D Vohra HA Sheppard MN Goldstraw P. Unusual sclerosing haemangiomas and sclerosing haemangioma-like lesions, and the value of TTF-1 in making the diagnosis. Histopathology. (2002) 41:404–13. 10.1046/j.1365-2559.2002.01522.x 12405908

[16] Gao Q Zhou J Zheng Y Cui J Teng X. Clinical and histopathological features of pulmonary sclerosing pneumocytoma with dense spindle stromal cells and lymph node metastasis. Histopathology. (2020) 77:718–27. 10.1111/his.14159 32441345

[17] Tanaka I Inoue M Matsui Y Oritsu S Akiyama O Takemura T. A case of pneumocytoma (so-called sclerosing hemangioma) with lymph node metastasis. Jpn J Clin Oncol. (1986) 16:77–86. 3009921

[18] Spencer H Nambu S. Sclerosing haemangiomas of the lung. Histopathology. (1986) 10:477–87. 10.1111/j.1365-2559.1986.tb02499.x 3013747

[19] Kim MK Jang SJ Kim YH Kim SW. Bone metastasis in pulmonary sclerosing hemangioma. Korean J Intern Med. (2015) 30:928–30. 10.3904/kjim.2015.30.6.928 26552471 PMC4642025

[20] Suzuki H Saitoh Y Koh E Hoshino H Kase D Kasei Y. Pulmonary sclerosing hemangioma with pleural dissemination: report of a case. Surg Today. (2011) 41:258–61. 10.1007/s00595-009-4220-5 21264765

[21] Wang X Zhang L Wang Y Jia X Wang J Zhang H. Sclerosing pneumocytoma with metastasis to the mediastinal and regional lymph nodes. Indian J Pathol Microbiol. (2018)61:407–9. 10.4103/IJPM.IJPM_98_17 30004067

[22] Teng X Teng X. First report of pulmonary sclerosing pneomucytoma with malignant transformation in both cuboidal surface cells and stromal round cells: a case report. BMC Cancer. (2019) 19:1154. 10.1186/s12885-019-6356-z 31775674 PMC6882242

[23] Jiang G Zhang M Tan Q Lin S Zeng Y Liu C. Identification of the BRAF V600E mutation in a patient with sclerosing pneumocytoma: a case report. Lung Cancer. (2019) 137:52–5. 10.1016/j.lungcan.2019.09.004 31546071

[24] Fan X Lin L Wang J Wang Y Feng A Nie L. Genome profile in a extremely rare case of pulmonary sclerosing pneumocytoma presenting with diffusely-scattered nodules in the right lung. Cancer Biol Ther. (2018) 19:13–9. 10.1080/15384047.2017.1360443 29236566 PMC5790398

[25] Tasci E Tezel C Orki A Akin O Falay O Kutlu CA. The role of integrated positron emission tomography and computed tomography in the assessment of nodal spread in cases with non-small cell lung cancer. Interact Cardiovasc Thorac Surg. (2010) 10:200–3. 10.1510/icvts.2009.220392 19933240

[26] Lv YL Yuan DM Wang K Miao XH Qian Q Wei SZ. Diagnostic performance of integrated positron emission tomography/computed tomography for mediastinal lymph node staging in non-small cell lung cancer: a bivariate systematic review and meta-analysis. J Thorac Oncol. (2011) 6:1350–8. 10.1097/JTO.0b013e31821d4384 21642874

[27] Miyagawa-Hayashino A Tazelaar HD Langel DJ Colby TV. Pulmonary sclerosing hemangioma with lymph node metastases: report of 4 cases. Arch Pathol Lab Med. (2003) 127:321–5. 10.5858/2003-127-0321-PSHWLN 12653576

[28] Adachi Y Tsuta K Hirano R Tanaka J Minamino K Shimo T. Pulmonary sclerosing hemangioma with lymph node metastasis: a case report and literature review. Oncol Lett. (2014) 7:997–1000. 10.3892/ol.2014.1831 24944657 PMC3961406

